# Disparities in Physical Function and Rehabilitation Utilization: A Cross-sectional Study of Patients Hospitalized for Acute Medical Illness

**DOI:** 10.1007/s11606-025-09826-7

**Published:** 2025-09-18

**Authors:** Maylyn Martinez, Rachel Baccile, Joshua Johnson, Jason Falvey, Monica E. Peek, Vineet Arora, David Meltzer

**Affiliations:** 1 Section of Hospital Medicine, Department of Medicine, University of Chicago, Chicago, IL USA; 2 Center for Health and Social Sciences, University of Chicago, Chicago, IL USA; 3 Department of Surgery, Section of Orthopedic Surgery, Duke University School of Medicine, Durham, NC USA; 4 Department of Physical Therapy and Rehabilitation Science, University of Maryland School of Medicine, Baltimore, MD USA; 5 Pritzker School of Medicine, Department of Medicine, University of Chicago, Chicago, IL USA; 6 Pritzker School of Medicine, The University of Chicago, Chicago, IL USA

**Keywords:** Hospital medicine, Health disparities, Social determinants of health, Functional decline, Hospital-acquired conditions, Frailty, Physical therapy, Physical rehabilitation

## Abstract

**Background:**

Black patients have poorer physical function and mobility compared to White patients but utilize physical rehabilitation services less.

**Objective:**

To determine whether racial differences exist in physical function and post-acute care facility utilization and whether neighborhood social disadvantage influences these differences.

**Design:**

A cross-sectional study of hospitalized patients at a large urban academic hospital.

**Participants:**

We included self-identified Black or White adults who were hospitalized between January 2018 and May 2022.

**Main Measures:**

The main exposure was self-identified race. The exposure in our secondary aim was neighborhood social disadvantage measured by the Area Deprivation Index. Neighborhood disadvantage was categorized as “more” (deciles 6–10) vs. “less” (deciles 1–5). Primary outcomes included inpatient functional measures, inpatient and post-acute physical therapy referral, and discharge location.

**Key Results:**

Compared to White patients, Black patients had more functional impairment on admission [OR 1.52; 95% CI (1.37, 1.69)], poorer mobility change [β-0.83; 95% CI (− 1.16, 0.50)], more mobility loss [OR 1.25; 95% CI (1.12, 1.41)], but no statistically significant difference in inpatient PT referrals after adjusting for physical function. Most differences in function persisted when stratified by neighborhood disadvantage. Despite similar odds of recommendation for discharge to a PAC facility, Black patients had significantly lower odds of discharge to a facility [OR 0.53; 95% CI (0.42, 0.68). This difference persisted when stratified by more neighborhood disadvantage [OR 0.60; 95% CI (0.43, 0.85)] and less neighborhood disadvantage [OR 0.43; 95% CI (0.31, 0.61)].

**Conclusion:**

In this study of patients hospitalized at a large urban academic medical center, Black patients had poorer functional outcomes yet nearly half the likelihood of discharging to a post-acute care facility compared to White patients. Race was a more consistent driver of disparities than neighborhood disadvantage. Hospital, healthcare system, and public policy changes are needed to improve discharge location appropriateness and recovery after hospitalization for vulnerable patients.

**Supplementary Information:**

The online version contains supplementary material available at 10.1007/s11606-025-09826-7.

## INTRODUCTION

Hospitalization is an important precipitant of disability. Adults who are hospitalized are 60% more likely to develop long-term disability than those who are not^1^ and nearly one third of patients who are hospitalized for acute medical illness will be discharged with a new disabling functional impairment known as hospital-acquired disability (HAD).^2^ These patients have a threefold increase in risk of readmission^3^ and 29% will still have disability 1 year later^4^. Maintaining functional independence is strongly linked to quality of life and physical health, making it a top priority for patients.^5,6^ Those with unresolved HAD experience a threat to their independence^7^ and may require more healthcare resources over the long-term.^8,9^

While older age and baseline functional impairments are powerful drivers of HAD, whether race or social context affects risk remains unknown. Evidence suggests that Black patients and patients living in socially deprived neighborhoods may be at increased risk. For example, Black patients are more likely to develop mobility limitation, mobility loss, and functional decline than their White counterparts^11–16^ and living in a socially disadvantaged neighborhood can impair functional recovery after hospitalization.^17^ Early mobilization and physical therapy (PT) are important tools to treat and prevent HAD and functional impairment during hospitalization.^18–25^ For patients unable to care for themselves after hospitalization, admission to a post-acute care (PAC) facility may be required for safety and rehabilitation of activity and mobility limitations. However, despite their functional disadvantages,^11–16^ Black patients and those from socially deprived neighborhoods also have lower rates of PT referral. Disparities in PT utilization along the functional recovery pathway include the securing of placement in a PAC facility, in the amount and type of exercise prescribed, and with longer wait times between outpatient PT referral, appointment scheduling, and appointment date.^26–29^

Using the National Institute on Minority Health and Health Disparities (NIMHD) research framework^30^ as a model, the mechanisms for these disparities include individual, interpersonal, community, and societal-level factors within behavioral domains (i.e., health behaviors), sociocultural and environmental domains (e.g., interpersonal discrimination, structural racism), and healthcare system domains (e.g., healthcare insurance, health literacy, patient-clinician relationship, healthcare quality and access). In addition, disparities in these areas may be driven primarily by race, social disadvantage, or a meaningful combination of both.

Given the significant contribution of hospitalization to the development of disability and continued challenges in achieving health equity, understanding how the social construct of race acts within larger institutional and environmental contexts to affect trends in HAD and PT utilization is of significant importance. This knowledge can help inform hospital policies and other programs to improve equity in functional outcomes among hospitalized patients. Specifically, understanding the individual contributors to these disparities could contribute to the development of HAD risk and discharge prediction and planning models. This is an important step toward the equitable and efficient triage of scarce hospital resources such as physical therapists, social workers, and case managers. Therefore, the aim of our study was to assess potential racial differences in physical function and physical rehabilitation referral and utilization in hospitalized patients. Our secondary aim was to determine whether neighborhood-level social disadvantage, as measured by the Area Deprivation Index, affects these potential differences.

## METHODS

### Study Design and Population

This was a cross-sectional study of patients admitted for acute medical illness at a tertiary academic medical center between January 1, 2018, and May 6, 2022. We excluded admission occurring between January 1, 2020, and June 30, 2020, to account for abnormal hospital operations related to the COVID-19 pandemic, including physical therapy workflows and discharge protocols, which would affect our outcomes of interest. Patients were excluded if they had a length-of-stay ≤ 48 h, if they had invalid admission or discharge mobility scores, if they lacked a home address that could be matched to their neighborhood disadvantage score, or if they were discharged to hospice or a psychiatric facility, or died during admission. This study was approved by the University of Chicago Institutional Review Board [IRB22-0656].

### Predictor Variables

#### Race

Patients self-reported their race at the time of presentation to the hospital. Options for race that were included in our study were Black/African American or White/Caucasian. We did not include other races or Hispanic ethnicity in the study due to very small percentages of these patients at our institution.

#### Neighborhood Social Disadvantage

We used the Area Deprivation Index (ADI) to estimate the social disadvantage in the neighborhood in which patients reported living at the time of admission to the hospital. The ADI uses census data to calculate a composite level of social vulnerability related to housing, employment, poverty, and education at the census block group level.^31,32^ The models categorize each census block group into a decile based on statewide comparison. We used the state decile rankings for Illinois from the 2020 (v3.2) ADI, which was constructed using the 2016–2020 5-year estimates from the US Census’ American Community Survey. Participants’ nine-digit zip codes at hospital admission were used for ADI linkage. For those without a 9-digit zip code, home address was used to perform geocoding and determine ADI. Because every cohort of patients has unique combinations of social determinants of health, applying existing ADI threshold effects from the literature can misinform or bias results. Therefore, to establish our ADI categories, we performed an exploratory analysis of ADI to determine a threshold effect specific to our cohort. Specifically, we compared a histogram of ADI Illinois state deciles among our cohort to a histogram of ADI state deciles in all of Illinois. We used this to determine a binary cutoff that captured any skewing of social disadvantage. While the state of Illinois was left-skewed (less disadvantage), our cohort was heavily right-skewed (more disadvantage), representing relative disadvantage in our cohort compared to the rest of the state. Therefore, we categorized ADI deciles 1–5 as “less neighborhood disadvantage” and 6–10 as “more neighborhood disadvantage.”

### Outcome Variables

#### Function

To determine patients’ physical function, we used the Activity Measure Post-Acute Care (AM-PAC) basic mobility short form score.^33^ The AM-PAC measures a patient’s difficulty with performing three basic movements (turning over in bed, sitting down/standing up from a chair with arms, going from lying down to sitting at edge of bed) and the amount of help needed to perform three basic movements (moving between bed and chair, walking in the hospital room, climbing three to five steps). In our hospital, the AM-PAC has been collected hospital-wide since 2014. Nursing staff perform assessments on admission and every nursing shift thereafter as part of normal hospital operations. The variable “admission mobility score” was defined as the first score for the index hospitalization that was recorded within 48 h of admission. The variable “mobility change” during hospitalization was calculated as (discharge AM-PAC–admission AM-PAC), with a discharge AM-PAC score required within 48 h of discharge. Functional impairment on admission/discharge was defined as a first AM-PAC raw score of ≤ 18 (*t*-scale score 43.63). This cutoff is predictive of discharge destination within 48 h of admission^34–36^, which is reliably (13 points) beyond the minimal detectable change at 95% confidence of 4.5 points.^37^ Additionally, a raw score of 18 suggests the patient has difficulty moving between a bed and chair and likely cannot walk in hallways^38^.

#### Inpatient Physical Therapy Referral

A patient was considered to have an inpatient PT referral if there was an order placed for PT evaluation or PT treatment any time during admission.

#### Discharge Recommendation

PAC facility recommendation was based on physical and occupational therapist evaluation and was available in the electronic medical record (EMR). At each visit with the patient, the therapists use the results of mobility assessment to make a safe discharge recommendation including home without services, home with home health PT/OT, outpatient PT/OT, skilled nursing or sub-acute rehab facility, or acute inpatient rehabilitation. This variable was dichotomized into community (home without services, home with home health PT/OT, or outpatient PT) vs. PAC facility (skilled nursing facility, sub-acute rehabilitation, or acute inpatient rehabilitation facility). We used the final discharge recommendation for our analysis. We prioritized using the PT recommendation but if this data was missing, we used the OT recommendation given that our therapists come to a consensus before making a formal recommendation.

#### Discharge Disposition

Discharge disposition was dichotomized as described above for discharge recommendation (community vs. PAC facility) and was only assessed among patients who received a recommendation to discharge to a PAC facility.

### Covariates

Sex was dichotomized to male or female. Age at the time of hospital admission and length of stay (LOS) were treated as continuous variables. Insurance status was a categorical variable (private, public, or none). All data were obtained from the EMR.

### Statistical Analysis

We compared baseline characteristics of Black and White patients using Pearson’s chi-square tests for binary variables and *t*-tests for continuous variables. We used sequential logistic regression to test the association of the primary predictor variable, race, on each outcome controlling for covariates. Because of existing evidence of racial differences in physical function, we constructed three models for each outcome to separate the effects of basic sociodemographics from those of physical function and/or receipt of physical therapy. Model 1 tested only the unadjusted predictor variable, race. Model 2 tested the predictor variable adjusted for additional sociodemographics (age, sex, and insurance status) and LOS. Model 3 tested the independent variable adjusted for sociodemographics, LOS, and physical function and/or receipt of physical therapy variables where appropriate. We used the fully adjusted model (model 3) to assess discharge recommendation and discharge to PAC facility stratified by neighborhood disadvantage (more vs. less) to determine whether this alters any effect of race on the outcomes. *p*-values < 0.05 were considered statistically significant. All analyses were done using Stata version 18.0.

## RESULTS

### Study Population Characteristics

Our study population included 12,952 unique admissions (CONSORT diagram available in Supplement 1). The cohort was composed primarily of Black patients (82.2%) who, compared to White patients, were statistically more likely to be younger (60.5 vs 62.1 years), be female (57.8% vs 47.0%), and reside in a neighborhood with more disadvantage (71.9% vs 31.4%) (Table 1). Black patients were more likely to have public insurance (90.8% vs 65.3%) and among those with public insurance they were more likely to have managed care plans (55.2% vs 35.7%). Black patients had worse function, compared to White patients, at hospital admission (46.5 vs 49.1) and discharge (48.6 vs 51.1).

**Table 1:** Patient Sociodemographic and Clinical Characteristics

	Black/African-American	White	*p*-value
*n*	10,643	2,300	
Demographic characteristics			
Mean age at admission (SD)	60.5 (18.0)	62.1 (16.3)	< 0.001
Over 65	4800 (45.1)	1137 (49.4)	< 0.001
Sex			
Female	6156 (57.8)	1082 (47.0)	< 0.001
Male	4487 (42.2)	1218 (53.0)	
Insurance			
None	2 (0.0)	0 (0.0)	< 0.001
Private	980 (9.2)	797 (34.7)	
Public	9661 (90.8)	1503 (65.3)	
Traditional	4326 (44.8)	982 (65.3)	< 0.001
Managed care	5335 (55.2)	521 (34.7)	
Social disadvantage			
Less	2992(28.1)	1578 (68.6)	< 0.001
More	7651 (71.9)	722 (31.4)	
Hospitalization characteristics			
Mean LOS, days (SD)	7.75 (7.53)	8.39 (9.65)	0.001
At admission:			
AM-PAC mobility score	46.5 (11.7)	49.1 (11.3)	< 0.001
Functional impairment	5184 (48.7)	907 (39.4)	< 0.001
At discharge:			
AM-PAC mobility score	48.6 (11.4)	51.1 (10.5)	< 0.001
Functional impairment	4006 (37.6)	685 (29.8)	< 0.001
Mobility change	2.12 (7.84)	1.98 (7.99)	0.44
Hospital-acquired impairment	465 (4.4)	115 (5.0)	0.20
Received PT consult	6876 (64.6)	1486 (64.6)	1.00
PT discharge recommendation			
Community	3625 (34.1)	868 (37.7)	< 0.001
Post acute care	2953 (27.7)	543 (23.6)	
Discharge Disposition			
Community	8263 (77.6)	1805 (78.5)	0.40
Post acute care	2380 (22.4)	495 (21.5)	

*AM-PAC* activity measure for post acute care, *PT* physical therapy

Raw AM-PAC scores are converted to *t*-scale scores

Social disadvantage was measured using Area Deprivation Index state deciles and was dichotomized into less (deciles 1–5) vs. more (deciles 6–10) disadvantage

### Function

Black patients were significantly more likely to have functional impairment at the time of admission in the unadjusted model [OR 1.46; 95% CI (1.33, 1.60)] (Table 2). After controlling for age, sex, length of stay, and insurance status, the odds increased [OR 1.52; 95% CI (1.37, 1.69)]. There was not a statistically significant difference between Black and White patients’ change in mobility from admission to discharge in the unadjusted model or model 2, controlling for basic demographics. Controlling for admission mobility score and receipt of a PT consult, Black race had a statistically significant negative effect on mobility change during hospitalization [β − 0.83; 95% CI (− 1.16, − 0.50)]. Black patients were as likely as White patients to have mobility loss in the unadjusted model and after controlling for basic demographics. In model 3, additionally controlling for function on admission and PT referral, Black patients were significantly more likely to have mobility loss as compared to White patients [OR 1.25; 95% CI (1.12, 1.41)]. Differences by social disadvantage can be found in Table 3. The full results of regressions modeling can be found in Supplement 2.

**Table 2:** Functional Impairment, Mobility Change, Mobility Loss, and PT Referrals for Black Patients as compared to White Patients: Sequential Regression Modeling

*N* = 12,942	**Functional impairment at admission** Odds ratio 95% CI	**Mobility change** Coefficient 95% CI	**Mobility loss** Odds ratio 95% CI	**PT referral** Odds ratio 95% CI
**Model 1**				
Unadjusted	1.46*** 1.33, 1.60	0.14 − 0.22, 0.50	1.04 0.96, 1.16	1.00 0.91, 1.10
**Model 2**				
Adjusted for basic demographics	1.52*** 1.37, 1.69	0.01 − 0.37, 0.38	1.09 0.98, 1.22	1.14* 1.02, 1.28
**Model 3**				
Adjusted for basic demographics + physical function/PT referral	–- –- –-	− 0.83*** − **1.16,** − **0.50**	1.25*** **1.12, 1.41**	0.96 1.02, 1.28

*PT* physical therapy

Bolded results represent a health disparity (a health difference that adversely affects disadvantaged populations in comparison to a reference population)

Model 2 adjusts for sex, age, length of stay, and insurance status; model 3 additionally adjusts for admission mobility score, mobility change, and/or PT referral where appropriate

^*^p < 0.05; **p < 0.01; ***p < 0.001

**Table 3:** Inpatient Outcomes for Black Patients as Compared to White Patients Stratified by Social Disadvantage

	**Functional impairment at admission** OR 95% CI	**Mobility change** Coefficient 95% CI	**Mobility loss** OR 95% CI	**PT referral** OR 95% CI
Less Disadvantage (*n* = 4570)	1.79*** 1.55 2.07	− 0.72** − 1.18 − 0.26	1.22** 1.07 1.39	0.91 0.80 1.04
More Disadvantage (*n* = 8373)	1.12 0.93 1.33	− 1.09*** − 1.64 − 0.55	1.40* 1.04 1.90	1.06 0.80 1.41

*PT* physical therapy

Bolded results represent a health disparity (a health difference that adversely affects disadvantaged populations in comparison to a reference population)

Adjusted for sex, age, length of stay, and admission mobility score, mobility change, and/or PT referral where appropriate

^*^p < 0.05; **p < 0.01; ***p < 0.001

### Inpatient Physical Therapy Referrals

There was no difference between rates of PT referrals between Black and White patients in the unadjusted model. After controlling for demographics in model 2, Black patients were more likely to receive a PT consult [OR 1.14; 95% CI (1.02, 1.28)] as compared to White patients, but this difference was eliminated after adjusting for admission mobility score and change in mobility (Table 2). There were no differences in PT referrals when stratified by social disadvantage (Table 3).

### Recommendation for Discharge to PAC

In unadjusted analysis, Black patients had greater odds of receiving a recommendation for discharge to PAC [OR 1.30; 95% CI (1.16, 1.46)]. After controlling for basic sociodemographics, the odds remained higher for Black patients [OR 1.36; 95% CI (1.19, 1.56)], but after adjusting for physical function, Black and White patients had the similar odds of receiving a recommendation to discharge to a PAC facility (Table 4). In the stratified analysis, Black patients living in neighborhoods with less disadvantage had *higher* odds than White patients living in those neighborhoods of receiving a recommendation for discharge to a PAC [OR 1.31; 95% CI (1.05, 1.63)]. There was not a statistically significant difference in recommendation for discharge to PAC between Black and White patients living in neighborhoods with more social disadvantage (Table 5).

**Table 4:** Odds of Recommendation for DC to PAC facility and DC to PAC Facility for Black Patients Compared to White Patients: Sequential Regression Modeling

	**Recommendation for PAC facility** (*n* = 7989) OR 95% CI	**Discharge to PAC facility** (*n* = 3496) OR 95% CI
**Model 1**		
Unadjusted	1.30*** 1.16, 1.46	0.51*** 0.41, 0.64
**Model 2**		
Adjusted for basic demographics	1.36*** 1.19, 1.56	0.58*** 0.46, 0.73
**Model 3**		
Adjusted for basic demographics + physical function/PT referral	1.08 0.93, 1.26	0.53*** 0.42, 0.68

*DC* discharge, *PAC* post-acute care

Bolded results represent a health disparity (a health difference that adversely affects disadvantaged populations in comparison to a reference population)

Model 2 adjusts for sex, age, length of stay, and insurance status; model 3 adjusts for basic demographics plus admission mobility score and mobility change during hospitalization

^*^p < 0.05; **p < 0.01; ***p < 0.001

**Table 5:** Discharge Outcomes for Black Patients as Compared to White Patients Stratified by Social Disadvantage

	**Recommendation for DC to PAC** OR 95% CI	**DC to PAC**^**†**^ OR 95% CI
Less Disadvantage (*n* = 4570)	1.31* 1.05 1.63	0.43*** 0.31 0.61
More Disadvantage (*n* = 8373)	0.89 0.71 1.11	0.60** 0.43 0.85

*DC* discharge, *PAC* post-acute care

Bolded results represent a health disparity (a health difference that adversely affects disadvantaged populations in comparison to a reference population)

Adjusted for sex, age, length of stay, insurance status, admission mobility score, and mobility change

^*^p < 0.05; **p < 0.01; ***p < 0.001

^†^Discharge to PAC was analyzed only among those who had a recommendation for discharge to a PAC facility yielding *n* = 855 for those with less disadvantage and *n* = 1579 for those with more disadvantage

### Discharge to PAC Facility

Among patients who were recommended for discharge to a PAC facility, Black patients had a decreased odds of discharge to PAC in all three models [fully adjusted model OR 0.53; 95% CI (0.42, 0.68)] (Table 4). This pattern of racial difference persisted whether Black patients lived in neighborhoods with more social disadvantage [OR 0.43; 95% CI (0.31, 0.61)] or less social disadvantage [OR 0.60; 95% CI (0.43, 0.85)] (Table 5).

## DISCUSSION

In this study of adults hospitalized at an urban academic medical center, we observed substantial disparities in patterns of physical function and PT utilization. Increased functional impairment among Black patients largely accounted for the racial differences in inpatient PT referrals and recommendations for discharge to PAC facilities as these odds were comparable between Black and white patients after adjusting for function. Despite their similar odds of a recommendation, Black patients were about half as likely to discharge to a PAC facility in all models. Disparities in functional outcomes persisted in nearly all models and regardless of level of social disadvantage. These findings suggest that of race and social disadvantage, race is a more consistent driver of disparities in function and where patients rehabilitate after discharge, though social disadvantage may create additional barriers for patients already marginalized by their racial identity. The patterns we found also imply that racial disparities in physical function do not stem from differences in PT referral rates.

To reduce disparities in hospital-acquired functional impairment and disability, we must improve the delivery of physical therapy and discharge planning for hospitalized patients. Black patients experience delays in PT referral and appointments^28^ and therapists recommend fewer exercises for Black patients even when they are like their White counterparts in every other characteristic and despite saying race does not affect recommendations^39^. These findings may help explain why Black patients had poorer function on discharge and more mobility loss in our study. Delayed inpatient PT referrals may decrease the number of PT sessions administered or allow function to worsen. Implicit bias or discrimination occurring during sessions may affect exercise duration or intensity, impair health literacy, or cause patient health behaviors and coping mechanisms to weaken therapy participation. Further, the responsibility of maintaining and improving patients’ function should be shared by the entire care team. Nursing staff play a pivotal role in HAD prevention by mobilizing patients with less severe impairments. Physicians and advanced practice providers can greatly influence the prioritization of mobility by the care team and the patient by regularly communicating the importance of mobility in achieving a full recovery. All of these factors span the NIMHD’s behavioral, sociocultural, and healthcare domains from the individual to the societal levels.

We may begin to address these disparities by increasing the number of underrepresented healthcare workers and implementing meaningful education in cultural humility for all healthcare workers. Additionally, hospitals should implement explicit protocols that include assessment of risk for HAD and functional decline. Assessments should account for evidence-based risk factors for poor functional outcomes such as age, baseline function, race, and social disadvantage. Results of assessments could then be used within 48 h of admission to triage patients to rehabilitation with a physical therapist, mobilization with nursing staff, or independent ambulation. To address patient-level barriers, future studies should aim to improve our understanding of how the hospital environment and past or present mistreatment by the healthcare system affect patient trust and participation during mobilization sessions. This may reveal additional strategies for improving therapy participation and functional outcomes.

Our study found disparities in discharges to PAC facilities, which may be rationalized by existing evidence. Black patients and those from socially disadvantaged neighborhoods are less likely to be admitted to high-quality nursing homes^40^ and nursing homes in areas with more social disadvantage have poorer nurse staffing ratios^41^. Further, Black patients, including those in our cohort, are more likely than their White counterparts to live in these neighborhoods^42–44^. Therefore, knowledge of the quality of more proximal nursing homes may partially explain the racial differences we observed in discharge to PAC facilities. Thus, to reduce these disparities, policies must target issues like facility quality, bed availability, and nursing home accountability.

### Limitations

Our results must be interpreted in light of some limitations. First, we conducted our analysis in a cohort of patients from a single institution, which limits its generalizability to other populations. This is especially true given our disproportionately high percentage of Black patients (82%). Second, we did not control for any comorbidities or severity of illness. This is important as these variables may affect a patient’s physical function, their need for physical rehabilitation, and how they respond to and tolerate rehabilitation. However, previous studies show health status and comorbidities are nearly colinear with physical function. When included in modeling that adjusts for physical function, as ours did, the effects of comorbidities tend to be minimal^45^. Last, the ADI Neighborhood Atlas used 2016–2020 census data at the time of our analysis, while our cross-section took place from 2018 to 2022. Therefore, patients admitted in 2021 and 2022 may have had addresses on file that did not reflect accurate census data or updated ADI, so neighborhood disadvantage is potentially inaccurate for these patients.

## CONCLUSION

In this study, we found considerable disparities in functional status and discharge to post-acute care facilities. The discrepancy between need for and utilization of physical rehabilitation for Black patients underscores persistent dysfunction and structural inequities in our healthcare system despite increasing awareness. The prioritization of access to high-quality acute and post-acute physical rehabilitation services is essential to successfully reduce the contribution of hospitalizations to functional dependence and disability in vulnerable populations.

## Supplementary Information

Below is the link to the electronic supplementary material.

Supplementary Material 1 (DOCX 157 KB)

## Data Availability

The datasets analyzed during the current study are not publicly available due HIPAA restrictions but are available from the corresponding author on reasonable request.
