# Supplementary material for: Disparities in Physical Function and Rehabilitation Utilization: A Cross-sectional Study of Patients Hospitalized for Acute Medical Illness
Source: J Gen Intern Med. 2025 Sep 18;41(11):3113–20. doi: 10.1007/s11606-025-09826-7 (PMC13493681; doi:10.1007/s11606-025-09826-7)
Supplement: Supplementary file 1 [file 11606_2025_9826_MOESM1_ESM.docx]

Supplementary information.

CONSORT Diagram

*Notes*: LOS: Length of Stay; AM-PAC: Activity Measure for Post Acute Care; ADI: Area Deprivation Index; PAC: Post Acute Care.

Supplement 2. Full results of sequential regression modeling

**Table 1: Functional Impairment on Admission for Black Patients as compared to White Patients: Sequential Regression Modeling**

| n = 12, 943 | **Model 1** | | **Model 2** | |
| --- | --- | --- | --- | --- |
|  | OR | 95% CI | OR | 95% CI |
| Black | 1.46*** | 1.33   1.60 | 1.52*** | 1.37   1.69 |
| Male |  |  | 0.84*** | 0.77   0.90 |
| Age |  |  | 1.05*** | 1.05   1.05 |
| LOS |  |  | 1.07*** | 1.06   1.08 |
| Public insurance |  |  | 1.69*** | 1.49   1.91 |

* p < 0.05, ** p< 0.01, *** p < 0.001

**Table 2: Change in Mobility for Black Patients as compared to White Patients: Sequential Regression Modeling**

| N = 12, 943 | **Model 1** | | **Model 2** | | **Model 3** | |
| --- | --- | --- | --- | --- | --- | --- |
|  | Coefficient | 95% CI | Coefficient | 95% CI | Coefficient | 95% CI |
| Black | 0.14 | -0.22    0.50 | 0.01 | -0.37    0.38 | -0.83*** | -1.16   -0.50 |
| Male |  |  | 0.06 | -0.21    0.34 | 0.37** | 0.12    0.61 |
| Age |  |  | -0.02*** | -0.02   -0.01 | -0.09*** | -0.10   -0.08 |
| LOS |  |  | -0.03* | -0.05   -0.00 | -0.12*** | -0.14   -0.09 |
| Public insurance |  |  | 0.38 | -0.04    0.81 | -0.57** | -0.93   -0.21 |
| Admission mobility score |  |  |  |  | -0.36*** | -0.38   -0.35 |
| Consult to PT |  |  |  |  | -1.34*** | -1.65   -1.04 |

PT = Physical Therapy

* p < 0.05, ** p< 0.01, *** p < 0.001

**Table 3. Mobility Loss for Black Patients as compared to White Patients: Sequential Regression Modeling**

| N = 12,943 | **Model 1** | | **Model 2** | | **Model 3** | |
| --- | --- | --- | --- | --- | --- | --- |
|  | OR | 95% CI | OR | 95% CI | OR | 95% CI |
| Black | 1.04 | 0.94    1.16 | 1.09 | 0.98    1.22 | 1.25*** | 1.12    1.41 |
| Male |  |  | 0.96 | 0.88    1.04 | 0.91* | 0.83    0.99 |
| Age |  |  | 1.02*** | 1.02    1.02 | 1.03*** | 1.02    1.03 |
| LOS |  |  | 1.03*** | 1.03    1.04 | 1.04*** | 1.03    1.05 |
| Public insurance |  |  | 0.98 | 0.86    1.12 | 1.12 | 0.98    1.28 |
| Admission mobility score |  |  |  |  | 1.06*** | 1.06    1.07 |
| Consult to PT |  |  |  |  | 2.31*** | 2.06    2.58 |

PT = Physical Therapy

* p < 0.05, ** p< 0.01, *** p < 0.001

**Table 4: Inpatient PT Referrals for Black Patients as compared to White Patients: Sequential Regression Modeling**

| N = 12, 943 | **Model 1** | | **Model 2** | | **Model 3** | |
| --- | --- | --- | --- | --- | --- | --- |
|  | OR | 95% CI | OR | 95% CI | OR | 95% CI |
| Black | 1.00 | 0.91    1.10 | 1.14* | 1.02    1.28 | 0.96 | 1.02    1.28 |
| Male |  |  | 0.92 | 0.85    1.00 | 0.98 | 0.85    1.00 |
| Age |  |  | 1.06*** | 1.06    1.06 | 1.04*** | 1.06    1.06 |
| LOS |  |  | 1.17*** | 1.15    1.19 | 1.13*** | 1.15    1.19 |
| Public insurance |  |  | 1.08 | 0.96    1.22 | 0.87* | 0.96    1.22 |
| Admission mobility score |  |  |  |  | 0.92*** | 0.02    0.03 |
| Change in mobility |  |  |  |  | 0.97*** | 1.02    1.28 |

PT = Physical Therapy

* p < 0.05, ** p< 0.01, *** p < 0.001

**Table 5. Inpatient Outcomes for Black Patients as Compared to White Patients Stratified by Social Disadvantage**

|  | **Less Disadvantage (n = 4,570)**  **OR**  **95% CI** | | | | **More Disadvantage (n = 8,373)**  **OR**  **95% CI** | | | |
| --- | --- | --- | --- | --- | --- | --- | --- | --- |
|  | **PT Referral** | **Delta Mobility** | **FI on admission** | **Mobility Loss** | **PT Referral** | **Delta Mobility** | **FI on admission** | **Mobility Loss** |
| **Black** | 0.91  0.80    1.04 | -0.72**  -1.18   -0.26 | 1.79***  1.55   2.07 | 1.22**  1.07    1.39 | 1.06  0.80    1.41 | -1.09***  -1.64   -0.55 | 1.12  0.93   1.33 | 1.40*  1.04    1.90 |
| **Male** | 1.00  0.90    1.12 | 0.20  -0.21    0.60 | 0.85*  0.75   0.97 | 0.96  0.87    1.07 | 0.92  0.79    1.07 | 0.47**  0.16    0.77 | 0.83***  0.76   0.92 | 0.81**  0.69    0.94 |
| **Age** | 1.04***  1.04    1.05 | -0.09***  -0.11   -0.07 | 1.04***  1.04   1.05 | 1.03***  1.02    1.03 | 1.04***  1.04    1.05 | -0.09***  -0.10   -0.08 | 1.05***  1.05   1.05 | 1.03***  1.02    1.03 |
| **LOS** | 1.14***  1.12    1.15 | -0.10***  -0.14   -0.06 | 1.06***  1.05   1.08 | 1.04***  1.03    1.05 | 1.12***  1.09    1.16 | -0.13***  -0.16   -0.10 | 1.07***  1.06   1.08 | 1.04***  1.03    1.05 |
| **Public insurance** | 0.85*  0.73    0.99 | -0.57*  -1.13   -0.02 | 1.66***  1.37   2.00 | 1.13  0.97    1.32 | 0.91  0.72    1.16 | -0.65**  -1.14   -0.17 | 1.72***  1.45   2.03 | 1.08  0.84    1.41 |
| **Admission mobility score** | 0.92***  0.92    0.93 | -0.37***  -0.39   -0.34 | --- | 1.06***  1.05    1.07 | 0.92***  0.91    0.93 | -0.36***  -0.38   -0.34 | --- | 1.06***  1.06    1.07 |
| **Change in mobility** | 0.97***  0.96    0.98 | --- | --- | --- | 0.96***  0.95    0.97 | --- | --- | --- |
| **Consult to PT** | --- | -1.11***  -1.61   -0.62 | --- | 2.20***  1.92    2.53 | --- | -1.45***  -1.84   -1.07 | --- | 2.53***  2.07    3.08 |

PT = Physical Therapy; FI = Functional Impairment

* p < 0.05, ** p< 0.01, *** p < 0.001

**Table 6: Odds of Recommendation for DC to PAC facility for Black Patients as Compared to White Patients**

| N = 7989 | **Model 1** | | **Model 2** | | **Model 3** | |
| --- | --- | --- | --- | --- | --- | --- |
|  | OR | 95% CI | OR | 95% CI | OR | 95% CI |
| Black | 1.30*** | 1.16   1.46 | 1.36*** | 1.19   1.56 | 1.08 | 0.93   1.26 |
| Male |  |  | 1.10 | 1.00   1.21 | 1.28*** | 1.15   1.44 |
| Age |  |  | 1.05*** | 1.04   1.05 | 1.03*** | 1.02   1.03 |
| LOS |  |  | 1.10*** | 1.09   1.11 | 1.08*** | 1.07   1.10 |
| Public insurance |  |  | 1.76*** | 1.48   2.09 | 1.45*** | 1.19   1.75 |
| Admission mobility score |  |  |  |  | 0.87*** | 0.86   0.88 |
| Change in mobility |  |  |  |  | 0.91*** | 0.90   0.91 |

LOS = length of stay

* p < 0.05, ** p< 0.01, *** p < 0.001

**Table 7. Odds of Discharge to PAC Facility for Black Patients as Compared to White Patients**

| N = 3496 | **Model 1** | | **Model 2** | | **Model 3** | |
| --- | --- | --- | --- | --- | --- | --- |
|  | OR | 95% CI | OR | 95% CI | OR | 95% CI |
| Black | 0.51*** | 0.41   0.64 | 0.58*** | 0.46   0.73 | 0.53*** | 0.42   0.68 |
| Male |  |  | 1.37*** | 1.18   1.59 | 1.42*** | 1.22   1.66 |
| Age_ |  |  | 1.01*** | 1.01   1.02 | 1.01* | 1.00   1.01 |
| LOS |  |  | 1.06*** | 1.04   1.08 | 1.06*** | 1.04   1.07 |
| Public insurance |  |  | 0.87 | 0.63   1.20 | 0.84 | 0.60   1.16 |
| Admission mobility score |  |  |  |  | 0.95*** | 0.94   0.96 |
| Change in mobility |  |  |  |  | 0.96*** | 0.95   0.97 |

LOS = length of stay

* p < 0.05, ** p< 0.01, *** p < 0.001

**Table 8. Discharge Outcomes for Black Patients as Compared to White Patients stratified by social disadvantage**

|  | **Less Disadvantage**  **(n = 4570)**  **OR**  **95% CI** | | **More Disadvantage**  **(n = 8373)**  **OR**  **95% CI** | |
| --- | --- | --- | --- | --- |
|  | **Recommendation** | **DC to PAC**  **(n = 855)** | **Recommendation** | **DC to PAC**  **(n = 1579)** |
| **Black** | 1.31*  1.05   1.63 | 0.43***  0.31   0.61 | 0.89  0.71   1.11 | 0.60**  0.43   0.85 |
| **Male** | 1.26*  1.04   1.52 | 1.40*  1.07   1.81 | 1.31***  1.14   1.50 | 1.42***  1.17   1.73 |
| **Age** | 1.03***  1.02   1.04 | 1.00  0.99   1.01 | 1.03***  1.02   1.03 | 1.01**  1.00   1.02 |
| **LOS** | 1.09***  1.07   1.11 | 1.03***  1.01   1.05 | 1.08***  1.07   1.09 | 1.07***  1.06   1.09 |
| **Public insurance** | 1.36  1.00   1.87 | 0.97  0.59   1.59 | 1.45**  1.14   1.86 | 0.77  0.50   1.20 |
| **Admission mobility score** | 0.86***  0.85   0.88 | 0.95***  0.93   0.97 | 0.87***  0.86   0.88 | 0.95***  0.94   0.96 |
| **Change in mobility** | 0.90***  0.89   0.91 | 0.96***  0.94   0.98 | 0.91***  0.90   0.92 | 0.96***  0.95   0.98 |

DC = discharge; PAC = post-acute care; LOS = length-of-stay

* p < 0.05, ** p< 0.01, *** p < 0.001
